# Superior Mesenteric Vein Thrombosis: A Case Report Highlighting the Importance of Diagnostic Challenges and Successful Medical Management

**DOI:** 10.7759/cureus.109837

**Published:** 2026-05-28

**Authors:** Francisco Mercado, Johnnie Saifa-Bonsu

**Affiliations:** 1 Medicine, Tripler Army Medical Center, Honolulu, USA; 2 Internal Medicine, Tripler Army Medical Center, Honolulu, USA

**Keywords:** apixaban, computed tomography abdomen, diagnostic testing, superior mesenteric vein thrombosis, thrombophilia work-up

## Abstract

Superior mesenteric vein thrombosis (SMVT) is a rare intestinal condition associated with potentially devastating outcomes if not promptly diagnosed and treated. This condition presents with nonspecific or atypical symptoms, making early recognition challenging and often leading to delays in management. This study discusses a 47-year-old physically active male with seminoma in remission in the past, who presented with abdominal bloating and pressure. Computed tomography (CT) revealed extensive SMVT. A heparin drip was initiated and later switched to apixaban, resulting in complete resolution of the abdominal pain and stabilization of the thrombus. This case report underscores the importance of recognizing unusual presentations of SMVT, highlights the vital role of advanced imaging techniques, such as contrast-enhanced CT, in accurate diagnosis, and demonstrates the effectiveness of anticoagulant therapy in successful medical management.

## Introduction

Superior mesenteric vein thrombosis (SMVT) is a rare intestinal ischemic condition that accounts for approximately 5-15% of all mesenteric thromboembolic events and often leads to significant morbidity and mortality if not diagnosed and treated promptly [1]. SMVT has an estimated occurrence of approximately 2.7 cases for every 100,000 person-years, predominantly impacting individuals between the ages of 40 and 70 years, with a slight inclination toward males [2,3]. The incidence of SMVT has increased over the past 40 years [4], likely due to improved detection with contrast-enhanced abdominal computed tomography (CT). Given its rarity, early diagnosis remains challenging owing to nonspecific clinical presentations and a lack of awareness among clinicians.

The presentation of SMVT is often nonspecific, posing a diagnostic challenge. Patients may present with nonspecific symptoms such as nausea, vomiting, diarrhea, and anorexia [5]. These symptoms overlap with those of other abdominal pathologies, making early diagnosis and treatment more difficult [6]. The most frequent symptom of SMVT is abdominal pain, which is often described as severe and out of proportion to the physical examination findings [1]. Although less common, some patients may present with hematemesis or melena, which can further complicate the clinical picture and delay the diagnosis [7]. Laboratory testing may indicate higher levels of white blood cells, the presence of anemia, and raised D-dimer levels, all of which are signs of thrombosis and inflammation [1].

Even though acute mesenteric vein thrombosis is uncommon, the mortality rate for those affected varies between 20% and 50% [8]. If left untreated, SMVT may lead to bowel infarction, resulting in tissue damage and necrosis [9]. As thrombosis progresses, SMVT can lead to hemorrhagic shock and multiple organ failure [10,11]. Owing to its high mortality rate, early diagnosis and appropriate management are crucial for reducing adverse outcomes [8].

We present the case of a 47-year-old male who came to the hospital with abdominal pain and was eventually found to have SMVT. This case underscores the challenges in diagnosis and the successful medical management of this uncommon condition.

## Case presentation

A 47-year-old male was transferred to our facility with two days of nonspecific abdominal pain, described as bloating and a sensation of his abdomen being pushed aside. He had no history of hypertension, diabetes, recent surgery, coronary artery disease, smoking, or chronic kidney disease. He had stage 1A seminoma treated with orchiectomy and radiation in 2013. His persistent, nonradiating abdominal pain was not associated with nausea, vomiting, chest pain, or gastrointestinal bleeding. The patient did not exhibit fever, chills, or other systemic symptoms. As a physically active man, he denied recent travel, trauma, venous thromboembolism (VTE), or a family history of VTE.

At the other hospital, he presented with a mildly distended abdomen and diffuse tenderness, which was worse in the right upper quadrant. Vital signs were normal, with a blood pressure (BP) of 132/84 mmHg, temperature of 98.3°F, heart rate (HR) of 93 beats/minute, respiratory rate (RR) of 16 breaths/minute, and oxygen saturation 98% in room air. Contrast-enhanced CT angiography of the abdomen (Figures 1, 2) demonstrated extensive thrombosis involving the superior mesenteric vein with extension to the splenic vein, associated with mesenteric edema, and no CT evidence of bowel ischemia or infarction. An intravenous heparin drip was initiated. As our center specializes in veterans’ care, he was transferred for further treatment.

**Figure 1:**
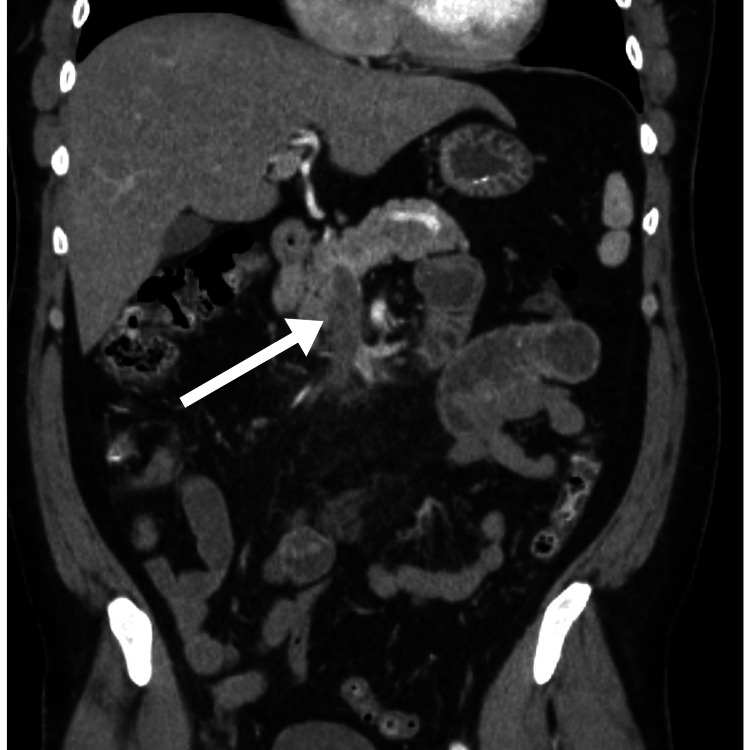
Initial coronal contrast-enhanced CT of the abdomen view showing an extensive SMVT that extends to the splenic vein, with mesenteric edema but no signs of bowel infarction (arrow). CT: computed tomography; SMVT: superior mesenteric vein thrombosis

**Figure 2:**
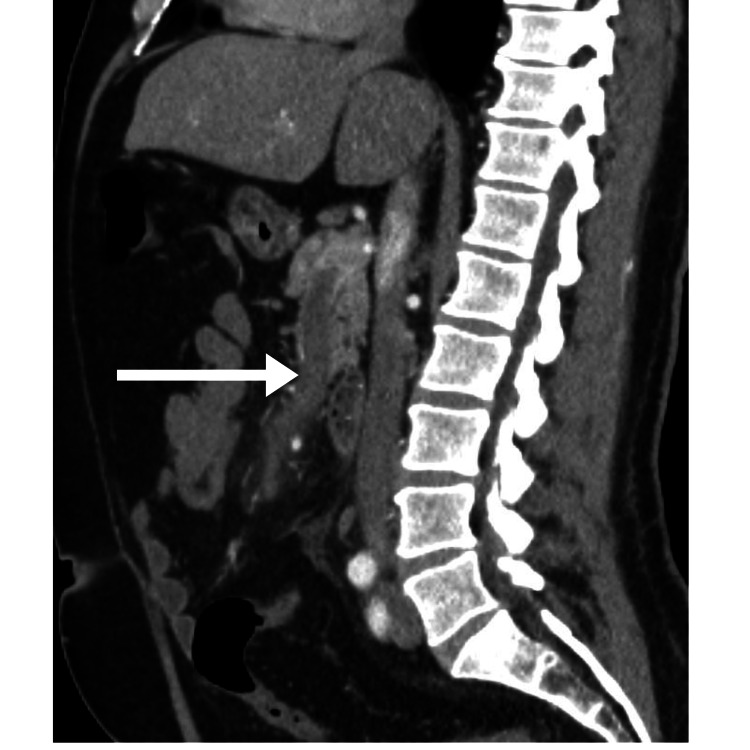
Initial sagittal contrast-enhanced CT of the abdomen showing an extensive SMVT with associated mesenteric edema with no bowel ischemia (arrow). CT: computed tomography; SMVT: superior mesenteric vein thrombosis

On the day of transfer, he was alert, well-oriented, and well-built. His vital signs were stable, with a temperature of 36.8°C, BP of 115/77 mmHg, HR of 80 beats/minute, RR of 16 breaths/minute, and body mass index (BMI) of 34.6 kg/m². Cardiovascular and pulmonary examinations were unremarkable. The abdomen was nontender, with no leg edema or asymmetry. The patient was monitored in the Progressive Care Unit. Upon admission, laboratory results indicated leukocytosis with a white blood cell (WBC) count of 11.3 × 10^9^/L. The hemoglobin level was normal at 14.4 g/dL, with platelets at 253 × 10^9^/L. Serum creatinine levels were normal at 1.2 mg/dL, and the lipase level was unremarkable at 31 U/L. Additionally, the lactic acid level was normal at 0.48 mmol/L. Table 1 presents a complete list of laboratory tests on admission. The heparin drip was continued at therapeutic doses (partial thromboplastin time goal of 60-90 seconds). Serial abdominal examinations performed every six hours revealed no recurrence of pain. The Vascular Surgery Team deemed thrombectomy or stent placement unnecessary, given the patient’s stability. Urology confirmed that his testicular cancer was in remission, with no mass on examination or CT, and normal tumor markers (beta human chorionic gonadotropin, alpha-fetoprotein, prostate-specific antigen) (Table 1). The hematology team advised transitioning to apixaban, beginning with 10 mg twice daily for one week, then reducing to 5 mg twice daily indefinitely, following a thorough evaluation that showed no genetic or acquired factors contributing to thrombotic risk (Table 2).

**Table 1:** Baseline laboratory values. WBC: white blood cell; PSA: prostate-specific antigen; HGB: hemoglobin; PLT: platelet; PT: prothrombin time; INR: international normalized ration; PTT: partial thromboplastin time; AST: aspartate aminotransferase; ALT: alanine transaminase; ALP: alkaline phosphatase

Laboratory parameter	Result	Reference
WBC	11.3 × 10^3^/µL (high)	3.5–10.4 × 10^3^/µL
HGB	14.4 g/dL	14.0–18.0 g/dL
PLT	253 × 10^3^/µL	140–440 × 10^3^/µL
Creatinine	1.2 mg/dL	0.9–1.3 mg/dL
PT	14.8 seconds	11.8–13.8 seconds
INR	1.10	0.8–1.1
PTT	26 seconds	21–34 seconds
AST	31 U/L	15–41 U/L
ALT	27 U/L	10–40 U/L
ALP	82 U/L	38–126 U/L
SARS-CoV-2 molecular	Negative	Negative
High-sensitivity troponin	3 ng/L	Under 22 ng/L
Alpha-fetoprotein	4.080 ng/mL	Under 10 ng/mL
PSA	0.630 ng/mL	Under 2.5 ng/mL

**Table 2:** Thrombophilia work-up. Ab: antibody; IgG: immunoglobulin G; GPL: immunoglobulin G (IgG) phospholipid units; MPL: immunoglobulin M (IgM) phospholipid units

Laboratory test	Result	Normal values
Anti-thrombin III	81%	80–130%
Factor V activity	92%	50–200%
Factor V Leiden mutation	No mutation found	-
Lupus anticoagulant	Not detected	-
Anticardiolipin Ab, IgG	Under 10 GPL	Under 15 GPL
Anticardiolipin Ab, IgM	Under 10 MPL	Under 13 MPL
Beta-2 glycoprotein IgA	<10 U/mL	Under 20 U/mL
Beta-2 glycoprotein IgG	<10 U/mL	Under 20 U/mL
Beta-2 glycoprotein IgM	<10 U/mL	Under 20 U/mL
Prothrombin gene mutation (G20210A)	No mutation found	-

The patient, who remained hemodynamically stable, was downgraded to the medical-surgical ward. His abdominal pain resolved, and he tolerated a regular diet. Follow-up CT abdomen with angiography before discharge showed no propagation of the thrombus. The patient was discharged in stable condition.

One month after admission, the patient showed no recurrence of abdominal pain and tolerated apixaban. The plan was to continue apixaban indefinitely because of the idiopathic SMVT. CT angiography at three months showed a decrease in the thrombus at the confluence of the superior mesenteric vein and splenic vein (Figures 3, 4). At six months, colonoscopy revealed benign polyps in the cecum and the transverse colon. He is currently on apixaban with ongoing hematology follow-up.

**Figure 3:**
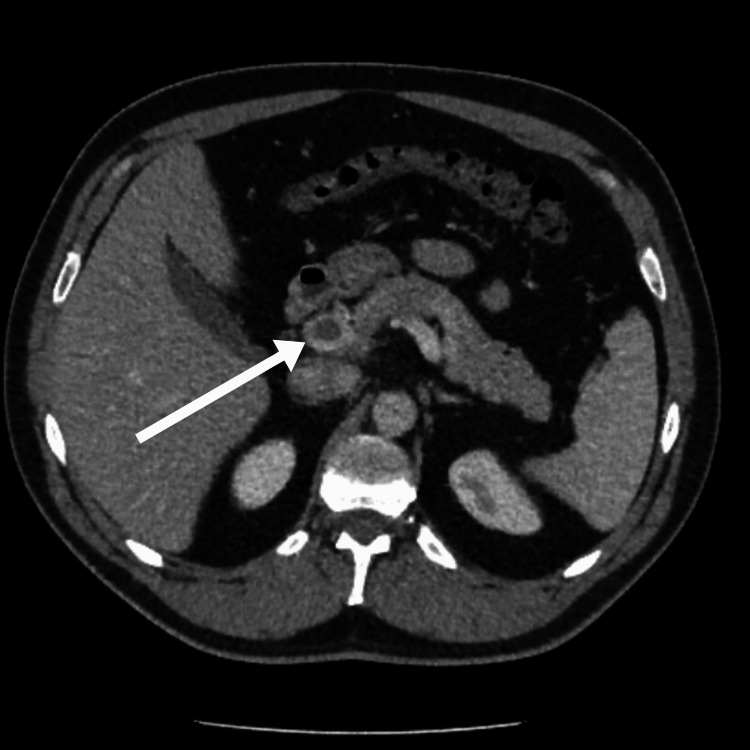
Axial contrast-enhanced CT image obtained at the three-month follow-up demonstrating interval decrease in the extent of SMVT (arrow). CT: computed tomography; SMVT: superior mesenteric vein thrombosis

**Figure 4:**
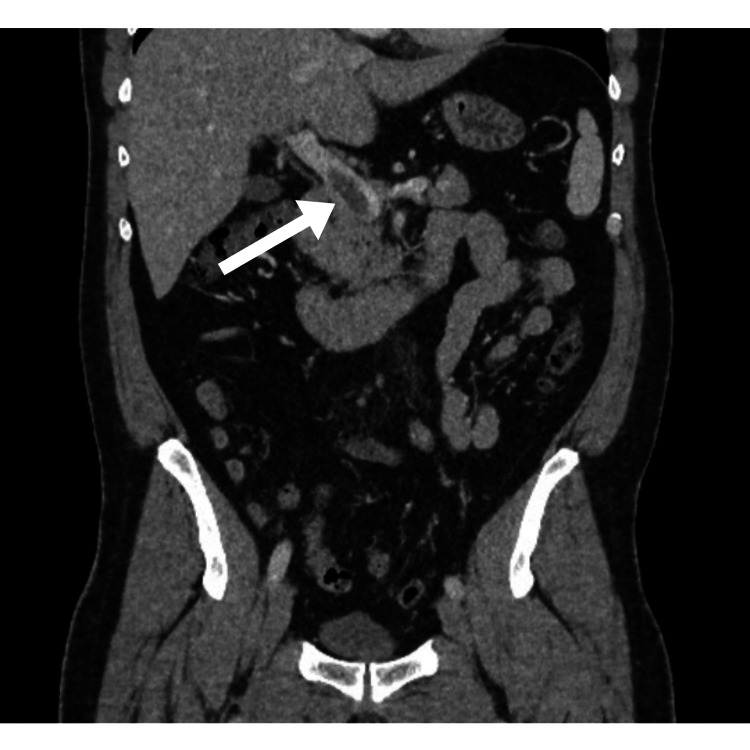
Coronal contrast-enhanced CT image at the three-month follow-up demonstrating interval decreased size of SMVT (arrow). CT: computed tomography; SMVT: superior mesenteric vein thrombosis

## Discussion

Clinical presentation

This case report describes a 47-year-old man who initially presented with nonspecific abdominal bloating and pressure. Abdominal pain is the most common presentation and is often variable in intensity and characteristics. In case series studies, abdominal pain has been described as cramping or bloating, with pain out of proportion to the physical examination [3]. Less frequently, abdominal pain may be accompanied by nausea, vomiting, and diarrhea, and in severe cases, when bowel infarction ensues, the patient may have melena or hematochezia [3,8]. Our patient presented with atypical abdominal symptoms not explained by any other cardiopulmonary or infectious process (pulmonary embolism, infectious pneumonia and effusion, pancreatitis, biliary disorders, and acute coronary syndrome were ruled out). The patient was subsequently diagnosed with extensive SMVT. This highlights the diagnostic challenge of SMVT, as the initial symptoms are often vague and resemble other abdominal issues, leading to delays or missed diagnoses [3].

Pathophysiology and risk factors

Risk factors for SMVT include inherited and acquired coagulopathies (e.g., deficiencies of protein S, protein C, and antithrombin III, as well as mutations in Factor V and prothrombin) [12], malignancy, infection, inflammatory states (inflammatory bowel disease), surgery, and trauma [13]. However, up to 25% of cases may appear idiopathic, and a thorough evaluation may reveal occult causes, including myeloproliferative neoplasms and undiagnosed malignancy [14]. Although advanced germ cell tumors (>stage 1) are highly associated with thromboembolic events (including VTE and pulmonary embolus), our patient showed no clinical evidence of recurrence of his seminoma based on his regular outpatient surveillance (both radiologic checks every five years and malignancy marker test every two years) and current CT studies; however, his radiation exposure during treatment could be a possible risk factor for his SMVT [15]. His slight leukocytosis on admission, which was not supported by any infectious processes or inflammatory conditions, resolved after SMVT treatment. However, obesity and post-bariatric surgery have been shown to be risk factors for mesenteric vein thrombosis [16,17]. Specifically, a case report published in 2023 involved a patient with a history of Hashimoto’s thyroiditis, a strong family history of malignancy, and Class II obesity who demonstrated an extensive inferior SMVT [17]. Our patient had Class I obesity (BMI: 31.7 kg/m²), which could have contributed to the development of SMVT. This underscores the need to carefully consider all predisposing factors when diagnosing SMVT.

The pathophysiology of SMVT involves a complex interplay of factors that leads to thrombus formation within the superior mesenteric vein, resulting in impaired venous return and potential bowel ischemia [10]. The underlying pathophysiology involves components of Virchow’s triad (venous stasis, endothelial injury, and hypercoagulability), leading to increased hydrostatic pressure, decreased oxygen delivery, and the potential for bowel wall edema, ischemia, and necrosis, if untreated [9]. Although the pathophysiology of SMVT is well understood, the condition remains difficult to diagnose because of its nonspecific symptoms and the need for advanced imaging.

Diagnosis

Contrast-enhanced CT is the most reliable method for diagnosing SMVT. Due to the high sensitivity and specificity, it identifies mesenteric venous thrombosis in up to 90% of cases, often revealing thrombus extension, bowel wall thickening, and mesenteric edema [8,12]. A complete hypercoagulability workup (protein C and S deficiencies, Factor V Leiden mutation, prothrombin gene mutation G20210A, antiphospholipid antibody syndrome, and JAK2 V617 mutation) is essential, especially in patients without standard risk factors [10]. Complete blood count may show leukocytosis and elevated but nonspecific inflammatory markers (e.g., C-reactive protein) [10]. Lactic acid levels may be monitored to assess ongoing bowel ischemia [3]. Our patient’s lactic acid level was not elevated at presentation, indicating the absence of bowel ischemia. His initial mild leukocytosis most likely represented inflammation due to SMVT. His hypercoagulability workup was unremarkable (Table 2). In this patient, JAK2 testing could have been evaluated to complete his workup. His protein C and S levels will be checked in three to six months to avoid artificially low readings during acute thrombotic events or while on anticoagulant therapy.

Management

Immediate anticoagulation is the cornerstone of therapy and is recommended for all patients unless contraindicated [3]. Low-molecular-weight heparin (LMWH) is preferred for stable patients, while unfractionated heparin is preferred for unstable patients, followed by oral anticoagulants (warfarin or direct oral anticoagulants) [18]. In cases refractory to medical therapy, endovascular treatments, such as catheter-directed thrombolysis, thrombectomy, stenting, and transjugular intrahepatic portosystemic shunt, may be indicated, with technical success and primary patency rates exceeding 80% in recent case series. Surgery is reserved for peritonitis or bowel necrosis [3,18]. Our patient received intravenous heparin infusion instead of LMWH due to a significant thrombosis burden and the potential need for surgery. After achieving clinical stability, he was transitioned to apixaban, as recommended by the hematology service. The duration of anticoagulation therapy is variable (three months to indefinite) and is often dictated by the underlying cause and risk factors [18]. Given the absence of bowel ischemia and contraindications to direct oral anticoagulants, our patient will take apixaban indefinitely, given no obvious risk factors (except Class I obesity), as recommended by the hematology service [19].

## Conclusions

SMVT is a rare but fatal condition if undiagnosed and untreated. Its presentation is nonspecific and often mimics other abdominal pathologies, making early diagnosis challenging. We present the case of a 47-year-old man with extensive SMVT who benefited from an appropriate diagnosis using CT and successful anticoagulation. This case underscores the critical role of advanced imaging in the early detection of SMVT and highlights the efficacy of timely anticoagulation in preventing severe complications and improving patient outcomes.
